# Kaposi’s Sarcoma-Associated Herpesvirus Lytic Replication Is Independent of Anaphase-Promoting Complex Activity

**DOI:** 10.1128/JVI.02079-19

**Published:** 2020-06-16

**Authors:** Endrit Elbasani, Silvia Gramolelli, Thomas Günther, Ildar Gabaev, Adam Grundhoff, Päivi M. Ojala

**Affiliations:** a Translational Cancer Medicine Research Program, University of Helsinki, Helsinki, Finland; b Heinrich Pette Institute, Leibniz Institute for Experimental Virology, Hamburg, Germany; c Cambridge Institute for Medical Research, University of Cambridge, Cambridge, United Kingdom; d Department of Infectious Diseases, Imperial College London, London, United Kingdom; Northwestern University

**Keywords:** APC/C, CDC20, CDH1, KSHV, Kaposi's sarcoma-associated herpesvirus, LEC

## Abstract

The anaphase-promoting complex, or cyclosome (APC/C), is a large E3 ubiquitin ligase composed of 14 subunits. The activity of APC/C oscillates during the cell cycle to ensure a timely transition through each phase by promoting the degradation of important cell cycle regulators. Of the human herpesviruses, cytomegalovirus (HCMV) and Epstein-Barr virus (EBV) both impair the activity of APC/C during their lytic replication cycle through virus-encoded protein kinases. Here, we addressed whether the oncogenic Kaposi’s sarcoma-associated herpesvirus (KSHV) deregulates the activity of APC/C during the lytic replication cycle. To this end, we used the well-characterized iSLK.219 cell model of KSHV infection and established a new infection model of primary lymphatic endothelial cells (LECs) infected with a lytically replicating KSHV BAC16 mutant. In contrast to those of EBV and HCMV, the KSHV lytic cycle occurs while the APC/C is active. Moreover, interfering with the activity of APC/C did not lead to major changes in the production of infectious virus. We further investigated whether rereplication stress induced by the unscheduled activation of the APC/C-CDH1 complex affects the number and integrity of KSHV viral episomes. Deep sequencing of the viral episomes and host chromosomes in iSLK.219 cells revealed that, while distinct regions in the cellular chromosomes were severely affected by rereplication stress, the integrity of the viral episomes remained unaltered.

**IMPORTANCE** DNA viruses have evolved complex strategies to gain control over the cell cycle. Several of them target APC/C, a key cellular machinery that controls the timely progression of the cell cycle, by either blocking or enhancing its activity. Here, we investigated the activity of APC/C during the lytic replication cycle of KSHV and found that, in contrast to that of KSHV's close relatives EBV and HCMV, KSHV lytic replication occurs while the APC/C is active. Perturbing APC/C activity by depleting a core protein or the adaptor proteins of the catalytic domain, and hence interfering with normal cell-cycle progression, did not affect virus replication. This suggests that KSHV has evolved to replicate independently of the activity of APC/C and in various cell cycle conditions.

## Abstract (precis)

DNA viruses have evolved complex strategies to gain control over the cell cycle. Several of them target APC/C, a key cellular machinery that controls the timely progression of the cell cycle, by either blocking or enhancing its activity. Here, we investigated the activity of APC/C during the lytic replication cycle of KSHV and found that, in contrast to that of KSHV's close relatives EBV and HCMV, KSHV lytic replication occurs while the APC/C is active. Perturbing APC/C activity by depleting a core protein or the adaptor proteins of the catalytic domain, and hence interfering with normal cell-cycle progression, did not affect virus replication. This suggests that KSHV has evolved to replicate independently of the activity of APC/C and in various cell cycle conditions.

## INTRODUCTION

Kaposi’s sarcoma-associated herpesvirus (KSHV) is the etiological agent of Kaposi’s sarcoma (KS), a common malignancy in people infected with HIV (1). KS is an angiogenic tumor arising from the endothelium of KSHV-infected individuals and is composed of spindle cells whose transcriptional profile resembles that of KSHV-infected lymphatic endothelial cells (LECs) (2–7). Besides KS, KSHV infection is further associated with two lymphoproliferative disorders, primary effusion lymphoma and multicentric Castleman disease (8, 9). In infected cells, KSHV can exist in a latent or lytic state. In the latent state, only a limited number of viral genes are expressed and, among them, the function of the latency associated nuclear antigen (LANA; encoded by ORF73) is the best understood. LANA is crucial for viral latent replication as it tethers the viral episomes to the host chromosomes and recruits the origin recognition complex and the replication licensing factors to the KSHV latent origin of replication. This ensures that viral episomes are replicated along with the cellular DNA once during each cell cycle (10–14). The viral lytic cycle is marked by the initial expression of ORF50, a strong viral transcriptional activator, which triggers the expression of a large subset of KSHV lytic genes that enable viral DNA replication, virus particle assembly, and release of the progeny (15, 16).

The anaphase-promoting complex, or cyclosome (APC/C), is crucial for cell cycle progression, as it acts as efficient E3 ubiquitin ligase machinery that targets various cell cycle regulators for degradation in a timely manner. APC/C substrates include cyclins, such as CCNB1 and CCNA2, and other key regulators such as GMNN, CDC6, CDC20, AURKA, and AURKB (17, 18). In G_1_, APC/C is associated with CDH1 (FZR1), a coadaptor protein that enables APC/C to target a specific subset of substrates for degradation. This prevents cells from proceeding to S-phase and allows licensing factors to associate at the cellular origins of replication (17, 19).

At the onset of S-phase, APC/C is inactivated by EMI1, a potent inhibitor of APC/C-CDH1 that occupies the APC/C catalytic domain and acts as a pseudosubstrate (20, 21). As a result, the levels of GMNN increase and, by binding to the CDT1 licensing factor, GMNN prevents CDT1 from promoting origin relicensing in S-phase. This ensures that the genome is replicated only once per cell cycle, thus preventing rereplication. When EMI1 is depleted during S-phase, APC/C-CDH1 is activated and induces rapid GMNN and CCNA2 degradation, among other substrates. This in turn allows DNA licensing factors to reassemble at the newly replicated DNA to promote cellular DNA rereplication, leading to DNA instability and genomic stress (22, 23).

In G_2_, prior to mitosis, the CDH1 subunit is phosphorylated to facilitate its dislocation from the APC/C-CDH1 complex to allow APC/C to recruit CDC20, the mitotic coadaptor protein. APC/C-CDC20 specifically degrades mitotic regulators like CCNB1, NEK2A, and securin (18, 24). Using a similar strategy, HCMV utilizes the viral UL97 protein kinase (vPK) to phosphorylate and dislocate the CDH1 subunit within the G_1_ phase (25, 26). In addition, HCMV expresses UL21a to induce the degradation of two APC/C subunits, APC4 and APC5, leading to the destruction of the whole complex (27). As a result, HCMV infection causes an accumulation of APC/C substrates such as GMNN, CCNB1, CDC6, and securin (25, 26, 28–30). Recently, it was found that Epstein-Barr herpesvirus (EBV) BGLF4 vPK, expressed during the EBV lytic cycle and a homologue of the KSHV ORF36, could also block APC/C activity (31). However, whether blocking of the APC/C by the EBV BGLF4 contributes to an efficient EBV lytic cycle was not addressed.

To date it was not known if the close relative of EBV, KSHV, would also interfere with the APC/C activity during the lytic replication cycle. In this study, we show that, contrary to HCMV and EBV, KSHV does not interfere with or require APC/C activity, suggesting that the KSHV lytic cycle is independent of APC/C activity. We also investigated the effect of rereplication stress induced by an unscheduled APC/C activation due to EMI1 depletion in the rapidly dividing cell line iSLK.219. We found that while cellular chromosomes were markedly affected by rereplication stress, surprisingly, the KSHV viral episomes were resilient to rereplication and their numbers in relation to the host genome did not change.

## RESULTS

### LECs infected with the KSHV-Lyt mutant virus are a useful model to study KSHV lytic replication.

To study the effects of KSHV lytic replication on the function of APC/C in a primary and relevant cell type for the KSHV life cycle, we developed a lytic KSHV infection model using primary human LECs to complement the well-characterized and widely used model iSLK.219 cells (32). LECs were chosen because the transcriptional profile of KS spindle cells resembles that of the KSHV-infected LECs (5), and the lymphatic endothelial environment is uniquely permissive to spontaneous KSHV reactivation (33, 34). To enrich the population of infected cells undergoing lytic replication, we used a KSHV-BAC16 mutant engineered to undergo lytic replication upon infection.

**FIG 1:**
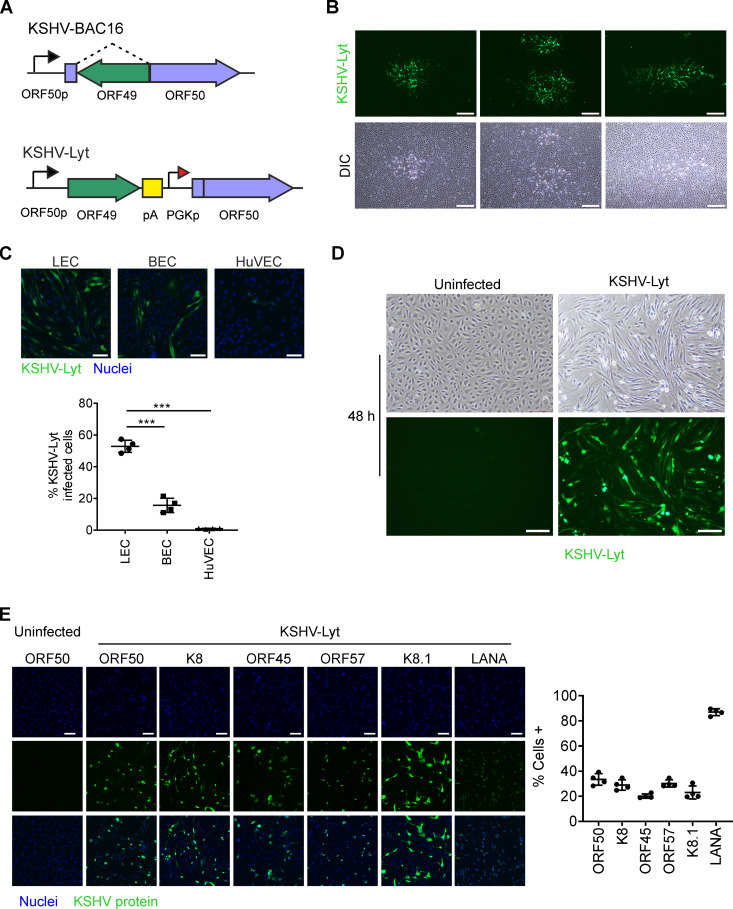
LECs infected with KSHV-Lyt mutant virus are a useful model to study KSHV lytic replication. (A) Schematic representation of the ORF50 region in the KSHV BAC16 and KSHV-Lyt mutant. (ORF50p, ORF50 promoter; PGKp, phosphoglycerate kinase 1 promoter; pA, polyadenylation signal) (B) Fluorescence and differential interference contrast (DIC) images of the KSHV-Lyt plaques appearing 2 weeks after KSHV-Lyt BAC transfection. Scale bar = 200 μm. (C) LECs, BECs, and HuVECs were infected with equal amounts of KSHV-Lyt. Upper panel: cells were stained with a GFP antibody and nuclei were counterstained with Hoechst 33342. Scale bar = 100 μm. Lower panel: quantification of the percentage of virus-infected cells. (*n* = 4 biological replicates; bars show SD; ***, *P* < 0.001). (D) DIC and fluorescence images of uninfected and infected LECs with KSHV-Lyt with 4 IU/cells at 48 h p.i. The KSHV-Lyt-infected cells express EGFP. (E) Staining of LECs infected with KSHV-Lyt (4 IU/cells at 48 h p.i.) with antibodies against LANA, ORF50, K8, ORF57, ORF45, and K8.1. Uninfected cells were stained with ORF50 antibodies. Representative images (left) and quantification of LECs expressing the indicated viral protein (right). Nuclei were counterstained with Hoechst 33342 (*n* = 4 biological replicates; bars show SD; scale bar = 100 μm).

The first lytic KSHV mutants were generated by M. Budt et al. (35), utilizing the KSHV-BAC36 backbone, and A. Gallo et al. (36), utilizing the KSHV-BAC16 backbone, by inserting a constitutively active promoter upstream of ORF50. We constructed a similar KSHV mutant utilizing the KSHV-BAC16, hereafter referred to as KSHV-Lyt in order to preserve the nomenclature suggested in the earlier studies (35, 36) (Fig. 1A). However, instead of reconstituting the virus using RPE-1 cells as done previously (35, 36), we reconstituted the KSHV-Lyt in LECs. Two weeks after the KSHV-Lyt DNA transfection, we detected the appearance of the first plaques, which continued to expand until all cells were infected (Fig. 1B). The virus released in the supernatant was used to infect LECs, human blood cells (BECs), and umbilical vein endothelial cells (HuVECs). At 72 h postinfection (p.i.), we quantified the KSHV-Lyt-infected cells using enhanced green fluorescent protein (EGFP), expressed by KSHV-Lyt, as a marker of infection. Strikingly, HuVECs were the most resistant, whereas LECs were the most susceptible to infection and BEC infection rates were three times lower than LECs (Fig. 1C). These results confirmed that LECs are a suitable, primary host cell model to study KSHV-Lyt replication.

To assess the expression of KSHV lytic proteins at the single-cell level, we infected LECs with four infectious units (IU)/cell. Using this infection dose, at 48 h p.i., the morphology of all cells changed from cobblestone to spindle cells (Fig. 1D), and at 96 h p.i., practically all cells rounded and detached from the bottom of the plate, indicating a cytopathic effect. We analyzed the expression of ORF50, K8, ORF45, ORF57, and K8.1 by immunofluorescence in the KSHV-Lyt-infected LECs at 48 h p.i., together with LANA, which is constitutively expressed during both viral latency and the lytic cycle. While LANA was expressed in almost all infected cells, the KSHV lytic proteins ORF50, K8, ORF57, ORF45, and K8.1 were expressed in about one third of the analyzed cells (Fig. 1E). This is in line with observations made in other experimental models such as BCBL1-TREX-RTA and iSLK.219 cells, where KSHV lytic replication (triggered by ORF50, expressed from a doxycycline-inducible promoter) occurs asynchronously and only in a subset of cells (32, 37, 38).

Overall, these findings show that LECs provide a highly permissive cellular environment for the KSHV-Lyt replication and are a suitable model to study the effects of KSHV lytic replication in primary cells.

### KSHV lytic replication occurs in G_1_ while APC/C is active.

**FIG 2:**
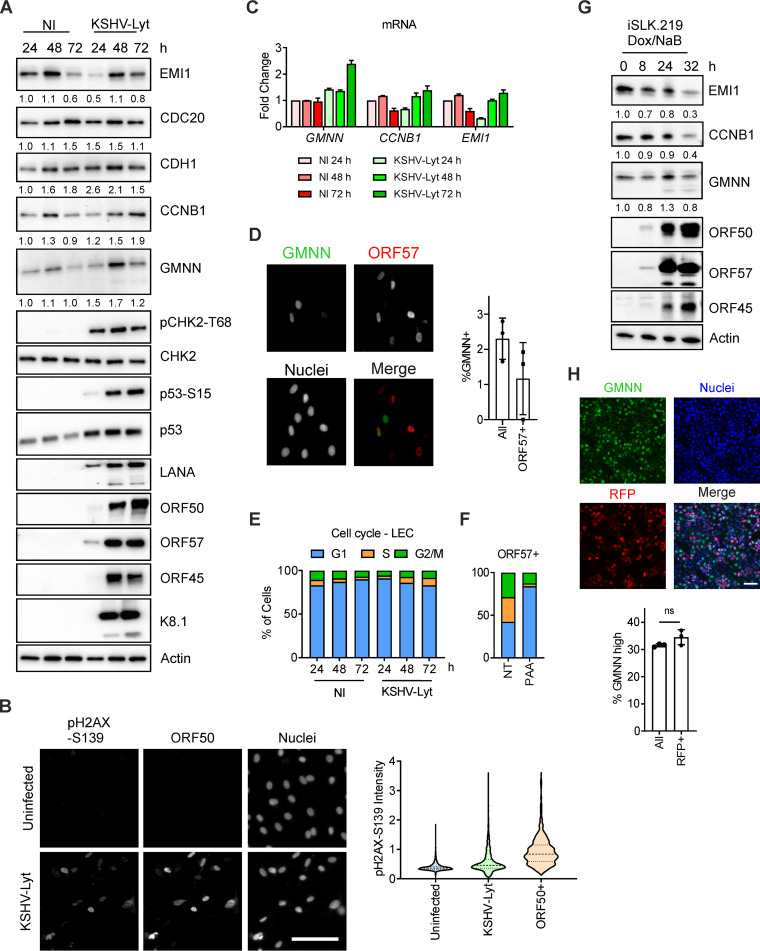
KSHV lytic replication occurs in G_1_ while the APC/C is active. (A) LECs were either infected with KSHV-Lyt using 4 IU/cell or uninfected. Cells were lysed at the indicated times and subjected to immunoblot analysis for the indicated proteins. Numbers below the blots indicate actin-normalized relative band intensity. (B) LECs were infected as in (A) or uninfected. At 48 h p.i., cells were stained with anti-ORF50 and anti-pH2AX-S139 antibodies. Quantification of the pH2AX-S139 intensity per nucleus is shown in the violin plot (*n* = >850 cells; scale bar = 100 μm). (C) LECs were infected as in (A) and harvested at the indicated time points. The mRNA levels of target genes were analyzed by RT-qPCR by using *Actin* mRNA as an internal control (*n* = 2 biological replicates; error bars show SD). (D) LECs were infected as in (A) and at 48 h p.i. stained with anti-GMNN and anti-ORF57 antibodies. The graph shows the percentage of the GMNN-positive cells in the total population and in the ORF57-positive population (*n* = 3 biological replicates; >500 cells/biological replicate were analyzed). (E) LECs were either infected with KSHV-Lyt as in (A) or uninfected. The cell cycle was analyzed by flow cytometry after PI staining. The graph shows the proportion of cells at each phase of the cell cycle. (F) LEC were infected as in (A), treated where indicated with PAA (100 μg/ml) and at 48 h p.i. stained with PI and antibodies against ORF57. The graph shows the portion of ORF57-positive cells at each phase of the cell cycle. (G) iSLK.219 cells were treated with Dox (1 μg/ml) and NaB (1.35 mM) to induce the KSHV lytic cycle for the indicated time. Immunoblot analysis of cellular lysates for the indicated proteins. Numbers below the blots indicate actin-normalized relative band intensity (H) iSLK.219 cells were treated with Dox and NaB as in (D) and at 24 h cells were stained with antibodies against GMNN. Nuclei were counterstained with Hoechst 33342. Representative images (upper panel) and quantification of GMNN-high cells in the total population and RFP-positive cells (lower panel) (*n* = 3 biological replicates; bars show SD; ns, *P* > 0.05; scale bar = 100 μm).

To test whether KSHV interferes with APC/C, we infected LECs with KSHV-Lyt using four IU/cell. Latent and lytic viral protein expression was confirmed by immunoblotting for LANA, ORF50, ORF57, ORF45, and K8.1 (Fig. 2A). Additionally, we noted that infection of LECs by KSHV-Lyt led to a marked increase in the levels of phosphorylated CHK2 at threonine 68 (pCHK2-T68) and p53 at serine 15 (pp53-S15) as early as 24 h p.i., marking the induction of the DNA damage response (DDR). The lytic cells, identified by high ORF50 expression, also showed higher levels of phosphorylated H2AX at S139 (pH2AX-S139), indicating the DDR was stronger in the lytic cells (Fig. 2A and B), as has been previously shown (39–41).

Next, we tested whether the APC/C was active during KSHV-Lyt infection by monitoring the levels of the APC/C adaptor proteins (CDH1 and CDC20), the APC/C inhibitor (EMI1), and two APC/C substrates (CCNB1 and GMNN), as compared to their levels in uninfected cells. Throughout the 72-h time course, we observed that both CDH1 and CDC20 were expressed in KSHV-Lyt-infected LECs, suggesting that both proteins are available as APC/C adaptors. The levels of CCNB1 did not increase markedly beyond the range that was observed in uninfected cells (<2-fold), while the GMNN levels fluctuated in a similar fashion as EMI1 (Fig. 2A), indicating that APC/C is functional during the KSHV lytic cycle. At the mRNA level, *EMI1* and *CCNB1* levels were downregulated at 24 h and later upregulated during the KSHV infection. In contrast, *GMNN* mRNA levels were either similar to uninfected or increased in the late stages of infection by 2-fold (Fig. 2C). Therefore, GMNN protein represents a suitable sensor of APC/C activity during the KSHV lytic cycle. In particular, accumulation of high GMNN levels in the nucleus indicates that the APC/C is inactive while lower levels of GMNN indicate that the APC/C is active. Further analysis of GMNN expression at the single-cell level showed that GMNN was expressed only in a small fraction of infected cells (2%). Among the lytic, ORF57-positive cells, only 1% of cells showed GMNN accumulation, indicating an inactive APC/C complex in these cells (Fig. 2D). Next, we analyzed the cell cycle profiles after staining the cellular DNA with propidium iodide (PI) and found that infected LECs accumulated mostly in G_1_, similarly to uninfected cells (Fig. 2E) and where APC/C-CDH1 is in an active state. The accumulation of cells in G_1_ was also observed when only the lytic cells (ORF57-positive) were analyzed after treatment with phosphonoacetic acid (PAA) to specifically block the KSHV genome replication and thus the accumulation of viral DNA in the nucleus (Fig. 2F). Altogether, the analysis of the APC/C substrate levels showed that APC/C activity in the KSHV-Lyt LECs (both in the total population and the ORF57-positive subset) is similar to the uninfected counterparts and the cell cycle profiles indicate that both of these KSHV-Lyt LEC populations accumulate in G_1_ where APC/C is active.

Subsequently, to validate our findings in another infection model, we repeated experiments using the cancer-derived iSLK.219 cell line stably infected with rKSHV.219, which constitutively expresses GFP under the control of the cellular EF1a promoter and RFP from the PAN promoter, an ORF50-responsive viral lytic promoter (42). In these cells the virus is in a latent state and lytic reactivation is induced through doxycycline (Dox)-inducible ORF50 expression (32). To induce the lytic cycle, we used, besides Dox, sodium butyrate (NaB), a histone deacetylase inhibitor commonly used to enhance KSHV reactivation (32). Here, we found that during KSHV reactivation, the total levels of APC/C substrates (GMNN and CCNB1) did not accumulate through the 32-h time course of the experiment but rather oscillated similarly to EMI1 levels (Fig. 2G). The expression patterns of ORF50, ORF45, and ORF57 in the lytic iSLK.219 cells were similar to KSHV-Lyt-infected LECs. We further investigated the oscillation of APC/C activity in lytic cells at the single-cell level using GMNN as a sensor. One day after inducing the lytic cycle, we found that the percentage of high-GMNN cells in the lytic (RFP-positive) subset of cells did not differ significantly from the whole-cell population (Fig. 2H). This shows that the GMNN levels oscillate in the same way in both the RFP-positive (lytic) and RFP-negative (latent) cells, and, consequently, suggests that APC/C functions are unaffected by the lytic replication cycle in iSLK.219 cells.

Taken together, these results show that during the lytic cycle in both the KSHV-Lyt-infected LECs and iSLK.219 cells, the APC/C remains under the control of its inhibitor EMI1 and retains the ability to degrade target substrates, such as GMNN.

### KSHV lytic replication cycle is independent from the APC/C activity.

Sequence analysis of the KSHV proteins (JSC1 strain; GenBank sequence GQ994935.1) using GPS-ARM software (43) revealed that several KSHV proteins (some of them essential for the completion of the lytic cycle) contain D-box motifs that can be recognized by the APC/C as a substrate for ubiquitination. D-box motifs were identified with high confidence in both viral kinases ORF21 (amino acids [aa] 51 to 54) and ORF36 (aa 9 to 12); in the early KSHV protein ORF45 (aa 289 to 291); and several components of the KSHV virion such as ORF19 (aa 284 to 287), ORF25 (aa 1242 to 1245 and aa 1335 to 1338), ORF26 (aa 234 to 237), and ORF65 (aa 71 to 74), therefore representing putative APC/C targets. Following this observation, we asked whether interfering with APC/C function to affect the ability to degrade targets would affect the KSHV lytic replication cycle. To this end, we assessed the levels of KSHV lytic proteins and infectious virus titers when APC/C-CDH1 was constitutively active (by EMI1 depletion) or inactive (by CDH1 depletion).

**FIG 3:**
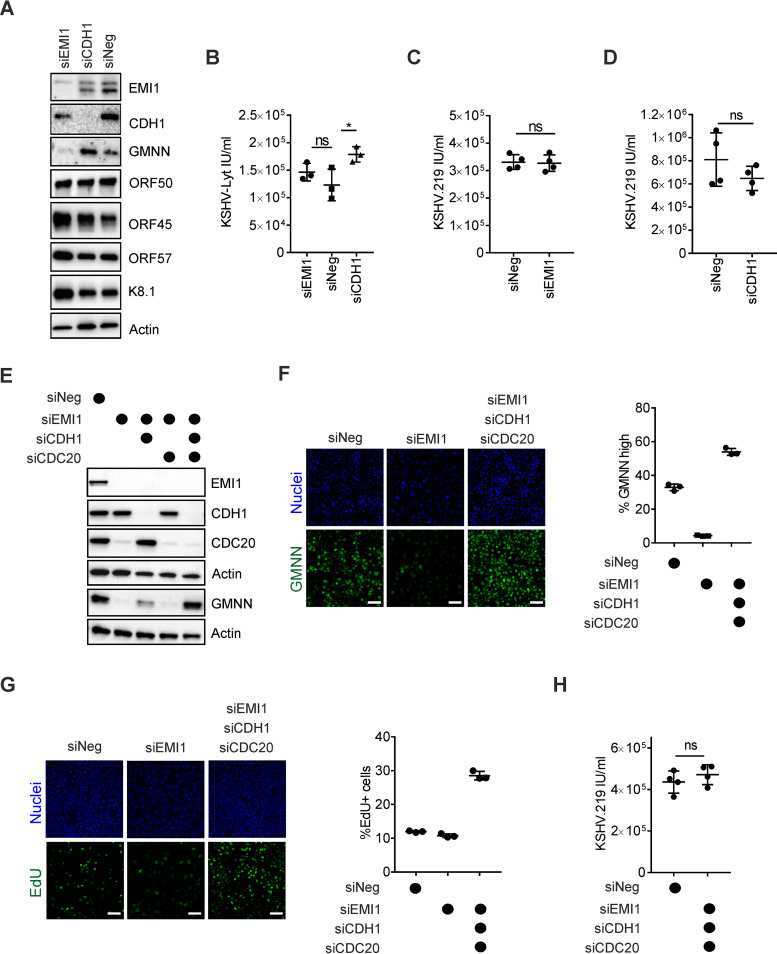
KSHV lytic replication cycle is independent of APC/C activity. (A) LECs were transfected with the indicated siRNAs (30 nM) and 24 h later infected with KSHV-Lyt (4 IU/cell). At 48 h p.i., cellular protein lysates were analyzed by immunoblotting for the indicated proteins. (B) LECs were treated as in (A) and at 72 h p.i., KSHV-Lyt titers were quantified (*n* = 3 biological replicates; bars show SD; *, *P* < 0.05; ns, *P* > 0.05). (C and D) iSLK.219 cells were treated with the indicated siRNA (10 nM) and with Dox (1 μg/ml) and NaB (1.35 mM). At 48 h, KSHV.219 titers were measured (*n* = 4 biological replicates; bars show SD; ns, *P* > 0.05). (E) Immunoblot analysis for the indicated proteins of iSLK.219 cells transfected with the indicated siRNA (10 nM) for 24 h. (F) iSLK.219 cells were transfected with the indicated siRNA (10 nM) and treated with Dox (1 μg/ml) and NaB (1.35 mM). At 24 h, the cells were stained with an antibody against GMNN. Representative images (left) and quantification of GMNN-high cells (right). Nuclei were counterstained with Hoechst 33342 (*n* = 3 biological replicates; bars show SD; scale bar = 100 μm). (G) iSLK.219 cells were treated as in (F). Two hours prior to fixation, the cell medium was further supplemented with EdU (10 μM). Representative images (left) and quantification of EdU-positive cells (right). Nuclei were counterstained with Hoechst 33342 (*n* = 3 biological replicates; bars show SD; scale bar =100 μm) (H) iSLK.219 cells were treated with the indicated siRNA (10 nM) and with Dox (1 μg/ml) and NaB (1.35 mM). Virus titers were measured at 48 h post reactivation (*n* = 4 biological replicates; bars show SD; ns, *P* > 0.05).

Depletion of CDH1 and EMI1, as well as the expression levels of KSHV lytic proteins, was confirmed by immunoblot in the KSHV-Lyt infected LECs (Fig. 3A). CDH1 depletion led to only slightly higher titers of KSHV-Lyt, while EMI1 depletion had no effect on the KSHV-Lyt titers in LECs (Fig. 3B). Next, we tested the effects of EMI1 and CDH1 depletion in the lytic iSLK.219 cells and found that the viral titers were comparable to the cells treated with the control small interfering RNA (siRNA) (Fig. 3C and D). This suggests that altering the APC/C-CDH1 activity does not affect the KSHV lytic cycle in either LECs or iSLK.219 cells.

Previous studies have shown that in actively dividing cancer cell lines, simultaneous depletion of CDH1 and CDC20 adaptor proteins, together with EMI1, i.e., three subunits of the APC/C, leads to a completely dysfunctional APC/C and a prolonged S-phase (19, 22, 44). We therefore measured the KSHV virus production from the reactivated iSLK.219 cells where CDH1, CDC20, and EMI1 had been depleted. Efficient silencing was verified by immunoblotting (Fig. 3E). Notably, upon EMI1 depletion, CDC20 levels also decreased dramatically, in line with CDC20 being both an adaptor protein and a substrate of the APC/C-CDH1. As an indication of efficient APC/C inactivation, we also monitored the levels of GMNN by immunoblotting and at the single-cell level. Immunoblot analysis of GMNN showed that in the absence of APC/C-CDH1, APC/C-CDC20 can degrade GMNN, although more inefficiently than the APC/C-CDH1 complex (Fig. 3E). Therefore, depletion of both CDH1 and CDC20 was required to completely inactivate the APC/C function. In cells where EMI1, CDC20, and CDH1 were depleted, the percentage of cells with high nuclear GMNN signal increased significantly compared to the siNeg-treated cells, whereas the siEMI1-treated cells, here used as a control, showed almost no cells with high nuclear GMNN levels (Fig. 3F). Next, upon EMI1, CDH1, and CDC20 depletion in iSLK.219 cells, we observed a 3-fold increase in the number of 5-ethynyl-2’-deoxyuridine (EdU)-positive cells (i.e., cells going/gone through S-phase) (Fig. 3G), suggesting a profound alteration of cell cycle regulation, as already reported for other cancer-derived cell lines (19, 44). However, when the KSHV titers in the reactivated iSLK.219 cells depleted of EMI1, CDH1, and CDC20 were measured, no significant differences were found compared to the control (siNeg-treated) cells (Fig. 3H). This suggests that the KSHV lytic cycle can occur efficiently in a cellular environment where the APC/C is inactive and encompassing a prolonged S-phase.

**FIG 4:**
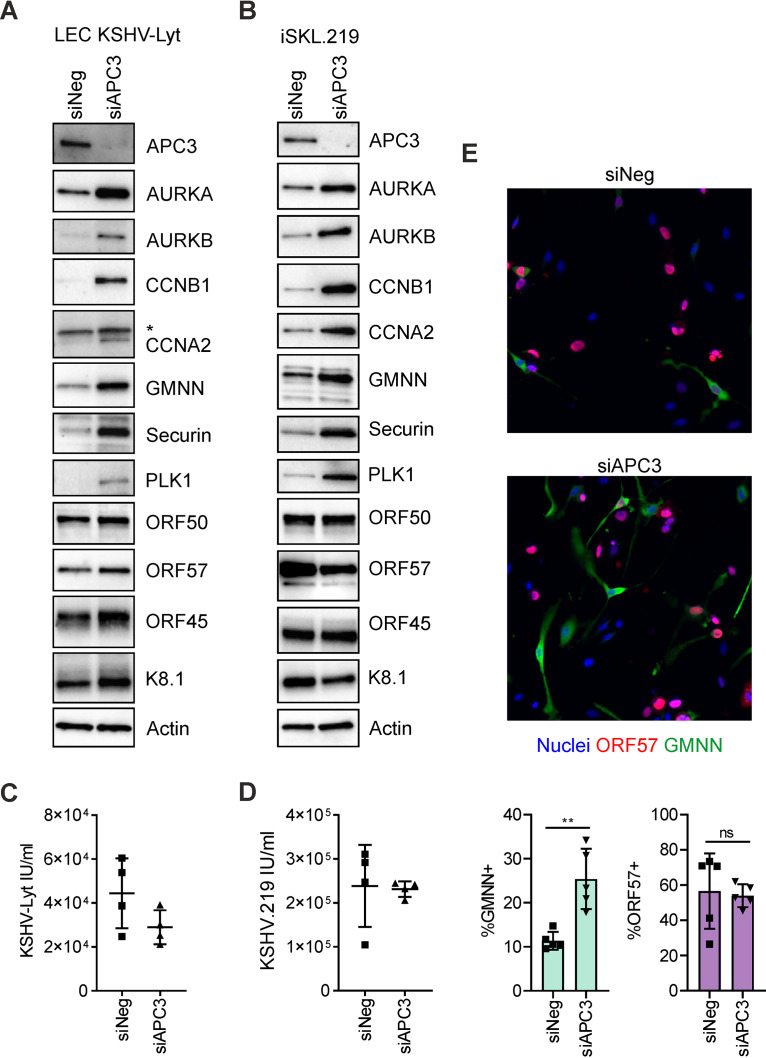
Inactivation of APC/C by APC3 depletion does not affect the KSHV lytic cycle. (A) LECs were transfected with the indicated siRNA (30 nM) and 24 h later infected with KSHV-Lyt (4 IU/cell). At 48 h p.i., cellular protein lysates were analyzed by immunoblotting for the indicated proteins. (B) Immunoblot analysis for the indicated proteins of iSLK.219 cells transfected with the indicated siRNA (10 nM) for 24 h and treated with Dox (1 μg/ml) and NaB (1.35 mM). (C and D) KSHV-Lyt titers (C) were measured at 48 h p.i. of the LECs in (A) and rKSHV.219 titers (D) were measured at 48 h post Dox/NaB induction of the iSLK.219 cells in (B) (*n* = 4 biological replicates; bars show SD). (E) LECs were transfected with the indicated siRNA (30 nM) and 24 h later coinfected with KSHV-Lyt and GMNN lentiviral expression vectors. At 48 h p.i., cells were stained with antibodies against ORF57 and GMNN. Representative images with nuclei counterstained with Hoechst33342 (upper panel) and quantification of ORF57 and GMNN expressing cells (lower panel) (*n* = 5 biological replicates; >250 cells/biological replicate; bars show SD; ns, *P* > 0.05; **, *P* < 0.01).

Similarly, depletion of APC3, an essential core protein of APC/C, increased the APC/C substrate levels (AURKA, AURKB, CCNB1, CCNA2, GMNN, securin, and PLK1) in both the KSHV-Lyt-infected LECs and iSLK.219 cells (Fig. 4A and B). Yet this did not influence the KSHV lytic cycle and virus titers (Fig. 4C and D). To obtain a better view of the effects of APC3 depletion in the KSHV-Lyt-infected LECs, we coinfected the siAPC3- and siNeg-treated KSHV-Lyt-infected LECs with a lentiviral vector expressing GMNN from the hCMV promoter, a constitutive strong promoter to enrich the population of GMNN-expressing cells. As expected, upon APC3 depletion, GMNN expression in the lytic cells (ORF57-positive) increased by more than 2-fold (Fig. 4E). Of note, we observed a cytoplasmic localization of GMNN in agreement with the study of M. Dimaki et al. (45), showing that when expressed by a constitutive promoter, GMNN is excluded from the nucleus and resides in the cytoplasm of G_1_ cells. Together these results show that disruption of the APC/C by depletion of one of its core proteins (APC3) does not affect the KSHV lytic replication cycle in either iSLK.219 cells or KSHV-Lyt LECs.

### Unscheduled APC/C activity-induced rereplication stress affects the integrity of cellular chromosomes, but not KSHV episomes.

**FIG 5:**
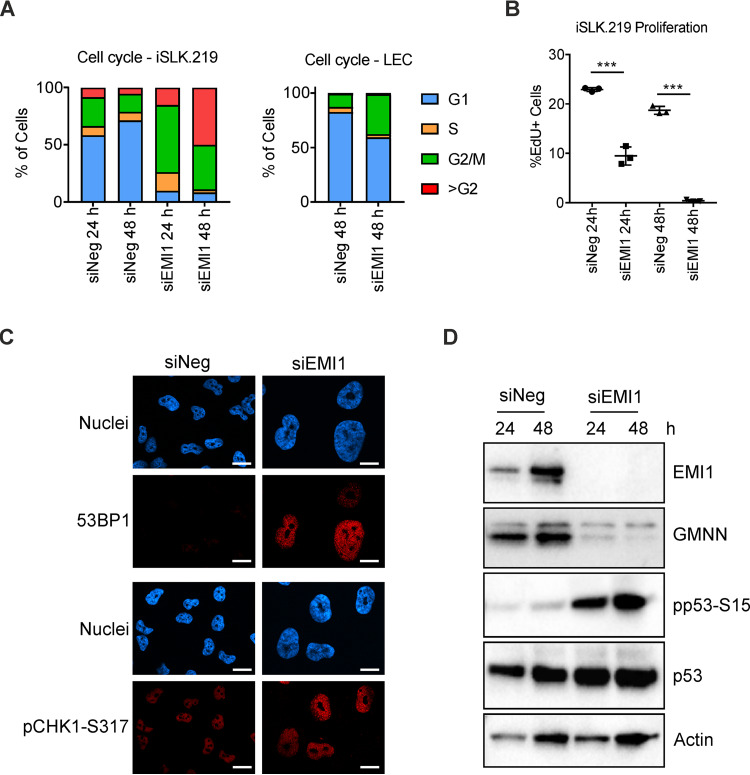
EMI1 depletion causes rereplication and genomic stress in iSLK.219 cells. (A) Latent iSLK.219 cells were treated with the indicated siRNA (10 nM) for 24 h and 48 h (left) while LECs were treated with the indicated siRNA (30 nM) for 48 h (right). Cell cycle profiles were obtained after PI staining by flow cytometry. (B) iSLK.219 cells were treated as in (A) and 2 h prior to fixation the cells were maintained in complete medium containing EdU (10 μM). Graph shows quantification of the EdU-positive cells at the indicated time points (*n* = 3 biological replicates; bars show SD; ***, *P* < 0.001). (C) iSLK.219 cells were treated for 48 h with the indicated siRNA (10 nM) and afterward stained with 53BP1 or pCHK1-S317 antibodies. Nuclei were counterstained with Hoechst 33342 (scale bar = 20 μm). (D) iSLK.219 cells were treated as in (A) and subjected to immunoblot analysis for the indicated protein targets.

In actively dividing cancer cells, in contrast to primary cells, one consequence of the unscheduled APC/C activation (due to EMI1 depletion) is the induction of rereplication stress (22, 23). Accordingly, when we compared the cell cycle profiles of primary LECs and the cancer-derived iSLK.219 cells after EMI1 depletion, we found rereplication occurring only in iSLK.219 cells (about 45% of the cells were >G_2_ at 48 h after EMI1 depletion), while most of the LECs remained in G_1_ with less than 1% of EMI1-depleted LECs undergoing rereplication (Fig. 5A). As a consequence of the rereplication-induced genomic stress in iSLK.219 cells, we found that the number of EdU-positive cells was reduced by half at 24 h and to less than 5% at 48 h after EMI1 depletion (Fig. 5B). Furthermore, the signals of 53BP1 and pCHK1-S317 in the EMI1-depleted iSLK.219 cells appeared punctuated (in foci) (Fig. 5C), and the levels of pp53-S15 increased as early as 24 h after EMI1 depletion (Fig. 5D), thereby confirming an ongoing DDR due to rereplication-induced genomic stress.

**FIG 6:**
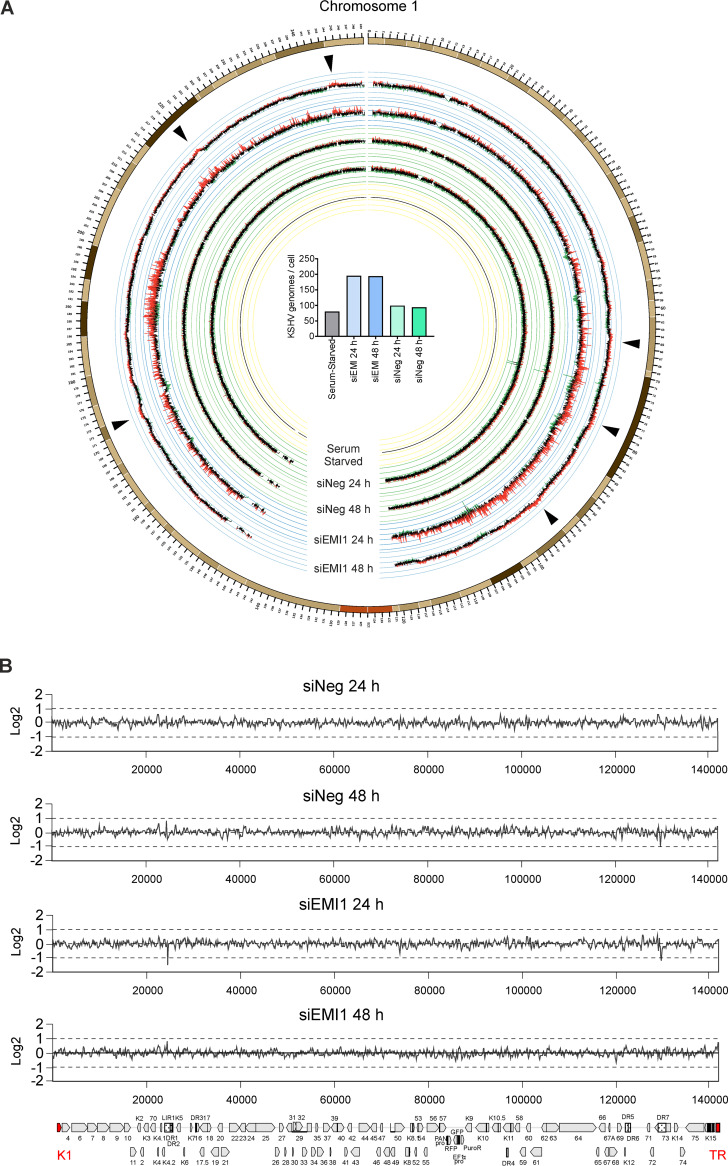
Rereplication stress following unscheduled APC/C activation affects the integrity of cellular chromosomes, but not KSHV episomes. (A) Latent iSLK.219 cells were treated with the indicated siRNAs (10 nM) or serum starved for 24 h. The genomic DNA of cells harvested at 24 and 48 h after siRNA treatment and of serum-starved cells was sequenced. Circos plot shows the read density distribution across human chromosome 1. Colors indicate log_2_ fold differences of >0.2 (red) and <−0.2 (green). Arrowheads show regions rereplicating at both 24 h and 48 h. In the center of the circos plot, the number of KSHV genomes per cell is shown. (B) Read density across the KSHV genome starting from the K1 to the terminal repeat (TR) region, both marked in red, and normalized to the serum-starved sample.

Interestingly, despite these deep alterations (i.e., the dramatic decrease in the EdU-positive cells and DDR induction), EMI1-depleted iSLK.219 cells were still able to sustain the full KSHV lytic replication cycle and released infectious virus to the same extent as the control (siNeg) treated cells (see Fig. 3C). Therefore, we hypothesized that rereplication stress in iSLK.219 cells would affect the cellular chromosomes but not the viral episomes. To test this, the genomic DNA of latent iSLK.219 cells undergoing rereplication (after EMI1 depletion) was subjected to next-generation sequencing and compared to the control treated cells (siNeg; Fig. 6A). To identify the rereplicating regions, the read densities across the KSHV genome and host chromosomes were analyzed. The genomic DNA isolated from serum-starved iSLK.219 cells to block proliferation was included to normalize the densities. In Fig. 6A, we show the densities across chromosome 1, as it is the largest chromosome and therefore expected to have the highest number of DNA replication origins. Remarkably, we found that after EMI1 depletion, multiple regions in this chromosome were rereplicated at both 24 h and 48 h post silencing (indicated by arrowheads in Fig. 6A). Additionally, we found that the number of KSHV episomes per cell doubled at 24 h post EMI1 depletion (Fig. 6A, center panel). Yet, the number of KSHV episomes per copy of the human genome was comparable to the control (siNeg) cells (at a ratio of approximately one), indicating that the KSHV episomes replicated in full synchrony with the host genome. However, there were no rereplicated regions at the latent origin of replication (K1 and TR regions, marked in red in Fig. 6B) or elsewhere across the entire KSHV genome, which explains why KSHV titers were not affected by the rereplication stress.

Altogether, these findings indicate that rereplication stress affects the integrity of the cellular genome but not the viral episomes, thus allowing the efficient production of infectious virus from the EMI1-depleted iSLK.219 cells.

## DISCUSSION

Several DNA viruses replicate efficiently in specific cell cycle phases. To enable this, viruses have evolved to target key cellular machineries such as the APC/C to gain control over cell cycle progression (46). In this study, we found that APC/C activity is not affected by the KSHV lytic replication cycle and the virus can replicate in the G_1_ phase where APC/C-CDH1 is active. This is in contrast to the other herpesviruses, HCMV and EBV, which have been found to block APC/C activity during their lytic cycle (25, 26, 29–31). Moreover, our data show that a functionally intact APC/C complex is dispensable for efficient KSHV reactivation. It is particularly interesting that blocking the APC/C function and thus dysregulating the cell cycle did not affect the KSHV lytic cycle. So, unlike other herpesviruses, which require specific cell cycle phases for efficient lytic replication, KSHV has adapted to reactivate and replicate efficiently in various cell cycle phases.

Besides G_1_, APC/C is also activated in response to DDR through the p53 signaling pathway, which leads to EMI1 downregulation (47). This, in turn, allows APC/C-CDH1 to trigger the degradation of important cellular regulators that promote S-phase transition and thereby facilitate the cell cycle block (47). KSHV lytic replication following virus reactivation and during the lytic burst that normally occurs upon primary infection leads to DDR (39–41, 48). In LECs *de novo* infected with KSHV-Lyt, the mRNA and protein levels of EMI1 and CCNB1 were initially downregulated but increased at later time points (48 and 72 h p.i) compared to uninfected cells. This could suggest that an intrinsic mechanism of KSHV during primary infection is to manipulate EMI1 levels in order to preserve the ability of the infected cells to proceed to S-phase while the virus establishes latency. Further supporting this hypothesis is the observation that GMNN mRNA is also upregulated during the KSHV lytic cycle (this study and reference 49), which is important for the progression of S-phase.

A recent study has shown that the KSHV-encoded K10 protein interacts with several subunits of the APC/C (50). Although we showed that KSHV lytic replication cycle does not interfere with the APC/C function, the utility of this interaction remains an open question. K10 is a late lytic protein (48 to 72 h) and localizes in the nucleus together with APC/C (38, 51). Interestingly, K10 has also been shown to interact with the CSL/CBF1 complex by occupying the binding site of Notch intracellular domain (NICD) and impairing NICD-mediated gene regulation (52). Possibly, binding to APC/C subunits may serve as a docking site for K10 to sequester the CSL/CBF1 complex away from chromatin.

K. J. Neelsen et al. (23) have shown that during rereplication the newly replicated DNA at the replication forks contains single-strand DNA gaps. Therefore, they predicted that rereplication of regions with DNA gaps leads to the accumulation of short double-stranded DNA fragments particularly encompassing the refiring replication origins. In our analysis of cellular chromosomes, the rereplicated regions were detected and these were more prominent at 24 h after EMI1 depletion when the cells were still able to undergo DNA replication. At 48 h post EMI1 depletion, when the cells stopped their DNA replication, the read densities across the cellular chromosomes normalized, suggesting that the short double-stranded DNA forms, predicted by K. J. Neelsen et al. (23), are short-lived.

A recent mapping of active and dormant origins of replication in HeLa cells indicated that active origins of replication localize in regions of open chromatin enriched with H3K9ac, H3K4me3, and H4K20me1 histone marks (53). In our analysis, we observed that the rereplicated regions were localized on specific sites of the chromosomes and not evenly distributed (Fig. 6A). Therefore, it is likely that the chromatin regions undergoing rereplication in iSLK.219 cells are more accessible (i.e., open) and contain higher densities of active replication origins.

KSHV episomes have been shown to contain at least two latent replication origins, the TR region (OriP) and the AT-rich region close to the K5 gene (OriA) (11, 14, 54, 55). Under normal conditions, KSHV replication can start at both origins and proceeds bi-directionally; however, there is a slight preference for OriP (56). Interestingly, the KSHV episomes were not affected by rereplication stress and replicated in full synchrony with the host DNA. It would be interesting in future studies to address how the KSHV origins of replication, one LANA-dependent (OriP) and the other LANA-independent (OriA), allow successful replication of KSHV episomes even during rereplication stress.

Our findings were confirmed in two different experimental systems, the iSLK.219 cells and LECs *de novo* infected with KSHV-Lyt, the latter providing a novel and useful model to study KSHV lytic replication in a physiologically relevant cell type. Overall, our results show that the KSHV lytic replication program occurs efficiently even when the cell cycle is strongly perturbed and is independent of the activity of APC/C.

## MATERIALS AND METHODS

### Cells.

Primary LECs (C-12216), BECs (C-12211), and HuVECs (C-12203), all from Promocell, were grown in Lonza EGM-2 (00190860) medium supplemented with EGM 2 MV Microvascular Endothelial SingleQuots (CC-4147). Vascular endothelial growth factor (VEGF) supplement was not added to the medium. In experiments, LECs, BECs, and HuVECs were used from passage 3 to 6. U2OS were obtained from ATCC (HBT-96) and iSLK.219 cells, generated by J. Myoung and D. Ganem (32), were a kind gift from Carolina Arias (University of California, Santa Barbara, US). U2OS, iSLK.219, and 293FT (Thermo Scientific) cells were propagated in Dulbecco’s modified Eagle medium (DMEM) (Sigma) supplemented with 10% fetal calf serum, 1% l-glutamine, and 1% penicillin/streptomycin. In the medium of iSLK.219 cells, puromycin (10 μg/ml) (Sigma), hygromycin B (600 μg/ml) (Sigma), and G418 (400 μg/ml) (Roche) were further supplied. To induce the KSHV.219 lytic cycle, iSLK.219 cells were treated with Dox (1 μg/ml) and NaB (1.35 mM). Phosphonoaceticacid (PAA) was obtained from Sigma.

### KSHV-Lyt BAC generation and reconstitution.

The design of the lytic KSHV BAC16 is based on the principle described for KSHV-Lyt BAC36 by M. Budt et al. (35). In this construct, the PGK promoter is inserted upstream of ORF50 (RTA) to enforce constitutive RTA expression. The lytic BAC16 was constructed as follows. In the first step, the shuttle vector containing the sequence of ORF49_KanR_pPGK was constructed using three fragments that were generated by PCR with overlapping primers as follows: (i) ORF49 was amplified from BAC16 (57); (ii) the KanR cassette with an I-SceI site was amplified from the pMCMV3 mRFP1 vector (kind gift of Martin Messerle); and (iii) the mPGK promoter was amplified from the pHRSIN vector. The fragments were cloned into a PstI/SalI-treated pMCMV3 mRFP1 vector by Gibson assembly. In the second step, the 2.6-kb fragment was amplified from the shuttle vector with primers containing arms of homology to BAC16, KSHV-Lyt forward primer 5′-ctccgcaaggggtagtctgttgtgagaatactgtccaggcagccacaaaaatgacatcgagaaggcccct-3′ and KSHV-Lyt reverse primer 5′-ccgagaggccgacgaagctttccacacaggaccgccgaagcttcttacccttgtcatcttgcgccatggt-3′. The resulting fragment was gel-purified and electroporated into GS1783 cells harboring BAC16 followed by a two-step traceless recombination procedure as described in B. K. Tischer et al. (58). The modifications were confirmed by restriction analysis and sequencing.

KSHV-Lyt virus was reconstituted by transfecting purified KSHV-Lyt BAC DNA in LECs using FugeneHD (Promega) at a DNA (μg) to FugeneHD (μl) ratio of 1:3.5 following the manufacturers recommendations. KSHV-Lyt spread to uninfected LECs was monitored by using EGFP, expressed by KSHV-Lyt, as a marker of infection. The supernatants containing KSHV-Lyt were stored at –80°C.

### Construction of GMNN lentivirus vector.

To construct the pLenti6.3-GMNN vector, the following primers were used to amplify the GMNN ORF from the cDNA of iSLK.219 and the pLenti6.3 backbone from the pLenti6.3-EGFP vector (provided by Genome Biology Unit Helsinki):

Fw 5′-TAGAGGATCCACTAGTCCAGTGTGGGCCACCATGAATCCCAGTATGAAGCAGAAACAAG-3′

Rv 5′-GTTAGGGATAGGCTTACCTTCGAACTCATATACATGGCTTTGCATCCGTAGAGG-3′

Fw 5′-GTTCGAAGGTAAGCCTATCCCTAAC-3′

Rv 5′-GGTGGCCCACACTGGACTAGTGGATC-3′

The purified PCR fragments were fused together using NEB HiFi DNA assembly MasterMix (NEB, E2611). The sequence of GMNN was verified by Sanger sequencing and the backbone integrity by restriction analysis.

### Lentivirus production.

pLP1, pLP2, and pVSVG (all from Thermo Scientific) together with pLenti6.3-GMNN lentiviral expression vector were transfected in 293FT cells using Lipofectamine 2000 (Thermo Scientific). The medium was replaced on the next day and harvested 72 h posttransfection. The virus-containing supernatant was filtered and mixed with 5× PEG-it (System Biosciences) and the virus particles were concentrated according to the manufacturer’s recommendations. Lentivirus titers were determined by infecting U2OS cells and by staining infected cells with GMNN antibodies.

### siRNA transfections.

Nontargeting siRNA (siNeg, L-005389) and siRNA targeting the human EMI1 (L-012434), CDH1 (L-015377), CDC20 (L-003225), and APC3 (L-003229) mRNA transcripts were purchased from Dharmacon. iSLK.219 and LEC cells were reverse transfected with 10 nM and 30 nM siRNA, respectively, using Lipofectamine RNAiMax (Thermo Scientific) according to the manufacturer’s recommendation. iSLK.219 cells were seeded at approximately 4 × 10^4^ cells/cm^2^ and LECs were seeded 2.5 × 10^4^ cells/cm^2^.

### Immunoblot analysis.

Cells were lysed in 1× Laemmli sample buffer. Proteins were separated using a 4 to15% Criterion TGX Precast midi gel (Bio-Rad) and subsequently transferred onto a Trans Blot Turbo midi nitrocellulose membrane (Bio-Rad). Protein transfer and loading on each membrane was assessed by Ponceau S staining. The membranes were blocked with 5% nonfat dry milk in TBS-T (Tris-buffered saline with 0.1% Tween) and were incubated with the indicated primary antibody diluted in 5% nonfat dry milk or bovine serum albumin (BSA) in TBS-T at 4°C overnight. The antibodies detecting GMNN (sc-13015), CDH1 (sc-166714), CCNB1 (sc-245), ORF57 (sc-135746), ORF45 (sc-53883), K8.1 (sc-65446), actin (sc-8432), APC3 (sc-9972), securin (sc-56207), CCNA2 (sc-271682), PLK1 (sc-17783), and p53 (sc-126) were from Santa Cruz Biotechnology (SCBT). EMI1 (37-6600) antibody was from Thermo Scientific, CDC20 (GTX5395) from GeneTex, AURKB (611082) from BD Biosciences, and ORF50 was a gift from Carolina Arias (UC Santa Barbara, USA). AURKA (3092), pp53-S15 (9284S), pCHK2-T68 (2197S), and CHK2 (6334S) antibodies were from Cell Signaling Technology (CST). LANA (ab4103) antibody was from Abcam. The membranes were further incubated with appropriate secondary antibody linked to horseradish peroxidase (HRP) diluted in 5% nonfat dry milk in TBS-T. Anti-rabbit IgG HRP-linked (7074S), anti-mouse IgG HRP-linked (7076S), and anti-rat IgG HRP-linked (7077S) secondary antibodies were from CST. The protein bands were revealed using WesternBright Sirius HRP substrate (Advansta) and images were acquired using a ChemiDoc gel imaging system (Bio-Rad).

Where indicated, band intensities were quantified using FIJI (59) software and shown in the figure as actin-normalized relative band intensities.

### Immunofluorescence analysis.

Cells were washed with phosphate-buffered saline (PBS) pH 7.4 and fixed in 4% paraformaldehyde in PBS, permeabilized using PBS with 0.2% Triton X-100 and blocked using PBS with 0.2% Triton X-100 containing 1% BSA. Cells were then incubated with antibodies against LANA (ab4103, Abcam), K8 (sc-69797, SCBT), ORF57 (sc-135746, SCBT), ORF45 (sc-53883, SCBT), K8.1 (sc-65446, SCBT), 53BP1 (NB100-305, Novus Biologicals), GMNN (sc-13015, SCBT), GMNN (ab195047, Abcam), pH2AX-S139 (05-636, EMD Millipore), pCHK1-S317 (number 12302, CST), ORF50 (a kind gift from Carolina Arias, UC Santa Barbara, USA), or GFP (a kind gift from J. Mercer, UCL, UK) where indicated. The cells were afterward incubated with appropriate secondary antibodies coupled to Alexa fluorophores. Anti-rabbit IgG (H+L) Alexa Fluor 488 (A-11034), anti-rabbit IgG (H+L) Alexa Fluor Plus 555 (A-32732), anti-rabbit IgG (H+L) Alexa Fluor 647 (A-32733), anti-mouse IgG (H+L) Alexa Fluor 647 (A-32728), anti-rat IgG (H+L) Alexa Fluor 647 (A-21247), and anti-mouse IgG (H+L) Alexa Fluor Plus 647 (A-32728) were acquired from Thermo Scientific. Nuclei were counterstained with 1 μg/ml Hoechst 33342 (Sigma) and the images were acquired using an AxioImager microscope equipped with an Apotome (Zeiss), ImageXpress Pico (Molecular Devices), or CellInsight High Content microscope (Thermo Fisher Scientific). The quantification and processing of images were done using the Cell profiler 3.0 (60) and FIJI (59) software packages.

### Cell cycle and proliferation analysis.

Cells were fixed with 70% ethyl alcohol (EtOH) and stained with antibodies against ORF57 (sc-135746, SCBT) and anti-mouse IgG (H+L) Alexa Fluor Plus 647 (A-32728, Thermo Scientific), where indicated. DNA was then stained with FxCycle PI/RNaseA staining solution (Thermo Scientific) for 1 h. The cells were analyzed using a BD Accuri C6 flow cytometer and data were processed using the CFlow Sampler software (BD).

For cell proliferation analysis, cells were maintained for 2 h in medium containing 10 μM 5-ethynyl-2’-deoxyuridine (EdU). Subsequently, the cells were fixed in 4% paraformaldehyde in PBS and the EdU incorporated in the cell DNA was coupled to Alexa Fluor 647 according to the manufacturer’s instructions provided in the Click-iT EdU Alexa Fluor 647 imaging kit (Thermo Fisher Scientific). Images were acquired using a CellInsight High Content microscope (Thermo Fisher Scientific) and analyzed using Cell Profiler 3.0 software to quantify EdU-positive cells.

### Reverse transcription-quantitative PCR analysis.

Cellular RNA was extracted using a NucleoSpin RNA extraction kit (Macherey-Nagel) and a total amount of 1 μg of RNA was reverse transcribed to cDNA using oligo(dT) primers and Multiscribe reverse transcriptase (Thermo Fisher Scientific) according to the manufacturer’s instructions. Equal amounts of cDNA were mixed with 2× SYBR reaction mix (Fermentas) together with appropriate dilution of the primer pairs specific for mRNA transcripts of *Actin* (5′-TCACCCACACTGTGCCATCTACGA-3′; 5′-CAGCGGAACCGCTCATTGCCAATGG-3′), *GMNN* (5′-ACAATGAAATTGCCCGCCTG-3′; 5′-GGTTCACCATCCAGTCTCTCT-3′), *CCNB1* (5′-GGTTGTTGCAGGAGACCATGTA-3′; 5′-CAGGTGCTGCATAACTGGAAG-3′) *EMI1* (5′-CCTCGACCCTCGGATAGTTGT-3′; 5′-TGTTGGGATATTGGTCTGGGC-3′). The RT-qPCRs were conducted in a Light Cycler 480 qPCR system (Roche).

### Virus titer determination.

To determine the KSHV-Lyt and rKSHV.219 titers, the supernatants were first serially diluted in appropriate media containing Polybrene (8 μg/ml, Sigma-Aldrich) and NaB (1.35 mM, Sigma-Aldrich). Afterwards, the virus dilutions were used to infect U2OS cells by spin infection (1,000 × *g*, 30 min, room temperature [RT]). The next day the cells were fixed and stained with an EGFP antibody (a kind gift from J. Mercer, UCL, UK) and the nuclei stained with Hoechst 44432 (1 μg/ml, Sigma). The cells were imaged using a CellInsight High Content microscope (Thermo Fisher Scientific) and quantified using the Cell Profiler 3.0 Software (60).

### DNA sequencing and data analysis.

Latent iSLK.219 cells were seeded at 4 × 10^4^ cells/cm^2^ and reverse transfected with the indicated siRNA (10 nM) for 24 h and 48 h or serum starved for 24 h. Cells from each sample (6 × 10^5^) were subjected to genomic DNA isolation using a NucleoSpin Tissue DNA extraction kit (Macherey-Nagel), including an RNase A treatment after proteinase K digestion. Additionally, prior to DNA isolation, bacteriophage Lambda DNA (N3011L, NEB) was added at 10 lambda genome copies/cell as a DNA spike-in control. The DNA sequencing was performed using NextSeq High Output system (Illumina), 1 × 75 bp single-end reads.

Single-end sequencing reads were aligned to the human reference genome (hg19), to the phage lambda reference sequence (GenBank NC_001416), and to the rKSHV.219 sequence, which is identical to the sequence of BAC16 (GenBank GQ994935), using Bowtie (61) with standard settings and the –m 1 option set to exclude multi-aligning reads. We inserted the panPromoter-RFP, GFP, and PuroR cassette, which are missing in the deposited GenBank sequence file, into the sequence of rKSHV.219 according to the original publication (42) before alignment. Read counting of viral ORFs was performed using featureCounts (62).

Coverage calculation and 0.1-kb window read counting for rKSHV.219 was performed with IGV-Tools (63). The resulting rKSHV.219 coverage data were normalized to the median of each respective data set. We then calculated the log_2_ fold change with respect to the control serum-starved sample. To assess coverage variations due to rereplication on the host chromosomes after treatment with siRNAs, we first generated normalized coverage tracks of 10-kb windows using Control-FREEC (64) and calculated the log_2_ ratios of all samples using the control sample (serum-starved) as the denominator, likewise to the calculations of rKSHV.219 coverage variation data. Circos plots of the resulting coverage variation tracks were then generated with Circos (65). Colors indicate log_2_ fold differences of >0.2 (red) and <−0.2 (green).

### Statistical test.

One-way ANOVA and Student’s *t* tests were carried out using GraphPad Prism 7 software. To test whether differences between specific means were significant, after one-way ANOVA, a Dunnett’s multiple-comparison test was used.

### Data availability.

Raw sequencing data are available in the Sequence Read Archive (BioProject PRJNA549967).
